# Prognostic Implications of the Complement Protein C1q in Gliomas

**DOI:** 10.3389/fimmu.2019.02366

**Published:** 2019-10-10

**Authors:** Alessandro Mangogna, Beatrice Belmonte, Chiara Agostinis, Paola Zacchi, Domenico Gerardo Iacopino, Anna Martorana, Vito Rodolico, Deborah Bonazza, Fabrizio Zanconati, Uday Kishore, Roberta Bulla

**Affiliations:** ^1^Department of Life Sciences, University of Trieste, Trieste, Italy; ^2^Human Pathology Section, Tumour Immunology Unit, Department of Health Sciences, University of Palermo, Palermo, Italy; ^3^Institute for Maternal and Child Health, IRCCS (Istituto di Ricovero e Cura a Carattere Scientifico) Burlo Garofolo, Trieste, Italy; ^4^Neurosurgical Unit, Department of Experimental Biomedicine and Clinical Neuroscience, University Hospital, Paolo Giaccone, University of Palermo, Palermo, Italy; ^5^Department of Health Promotion, Mother and Child Care, Internal Medicine and Medical Specialties, University of Palermo, Palermo, Italy; ^6^Department of Medical, Surgical and Health Science, University of Trieste, Trieste, Italy; ^7^Biosciences, College of Health and Life Sciences, Brunel University London, London, United Kingdom

**Keywords:** gliomas, C1q complement, bioinformatics analysis, survival probability, prognostic significance of C1q

## Abstract

The contribution of the complement system in the pathophysiology of brain cancers has been recently considered in light of its well-known involvement in carcinogenesis. Complement system represents an important component of the inflammatory response, which acts as a functional bridge between the innate and adaptive immune response. C1q, the first recognition subcomponent of the complement classical pathway, has recently been shown to be involved in a range of pathophysiological functions that are not dependent on complement activation. C1q is expressed in the microenvironment of various types of human tumors, including melanoma, prostate, mesothelioma, and ovarian cancers, where it can exert a protective or a harmful effect on cancer progression. Despite local synthesis of C1q in the central nervous system, the involvement of C1q in glioma pathogenesis has been poorly investigated. We, therefore, performed a bioinformatics analysis, using Oncomine dataset and UALCAN database in order to assess whether the expression of the genes encoding for the three chains of C1q (*C1qA, C1qB*, and *C1qC*) could serve as a potential prognostic marker for gliomas. The obtained results were then validated using an independent glioma cohort from the Chinese Glioma Genome Atlas datasets. Our bioinformatics analysis, coupled with immunohistochemistry and fluorescence microscopy, appears to suggest a positive correlation between higher levels of C1q expression and unfavorable prognosis in a diverse grade of gliomas.

## Introduction

The complement protein C1q represents the recognition subcomponent of the complement classical pathway, which is responsible for clearing immune complexes and invading pathogens. Its association with C1r and C1s, following ligand recognition, triggers complement activation (1, 2). C1q is characterized by a typical tulip-like overall structure, assembled from 18 polypeptide chains of three different types, A (28 kDa), B (25 kDa), and C (24 kDa), each having an N-terminal collagen-like domain and a C-terminal globular (gC1q) domain (3, 4). The gC1q domain, which is the ligand recognition region of C1q, has a heterotrimeric structure, being composed of C-terminal ends of A, B, and C chains (5). In addition to binding IgG and IgM containing immune complexes and activating the complement classical pathway, there is emerging evidence to suggest that C1q plays crucial roles in several processes that are independent of complement activation, such as placentation (6), angiogenesis (7), autoimmunity (8, 9), and carcinogenesis (9, 10). C1q is highly expressed in the microenvironment of various types of human tumors (10, 11) where it can exert either a protective or a detrimental effect on the tumor growth. In prostate cancer cells, C1q was recently shown to induce apoptosis by activating the tumor suppressor WOX1 (12), thus acting as an anti-tumor humoral factor. In ovarian cancer, C1q has been shown to induce apoptosis in a representative SKOV3 cell line via activation of TNF-α, upregulation of Fas, and downregulation of mammalian target of rapamycin, RICTOR, and RAPTOR survival pathways (13). In a BALB-neuT mouse model of mammary carcinomas, C1q was shown to have a protective role against cancer progression (14). However, C1q can promote adhesion, migration, and proliferation of primary cells derived from malignant pleural mesothelioma patients, a relatively rare disease associated with exposure to asbestos (11). This dichotomous role of C1q has been further highlighted by a bioinformatics analysis involving several types of carcinomas (15).

The importance of C1q in the pathophysiology of the central nervous system (CNS) has been an area of intense research in the last two decades. In a healthy brain, C1q promotes synapse elimination required for fine circuitry refinement during CNS development (16). C1q activities, unrelated to complement activation, were shown to support neuronal survival and neurite outgrowth *in vitro* and protect against β-amyloid-mediated neurotoxicity (17). C1q can also interact with abnormal protein aggregates, such as βA1-42, thus favoring neurodegenerative diseases progression (18).

Since C1q and other complement components can be locally produced within the CNS by microglia and astrocytes, it is likely that C1q has involvement in primary brain tumor pathophysiology (19). Brain malignancies arise from cells of the CNS and are classified according to the tissue of phylogenetic origin. Gliomas represent the most common and aggressive form of brain tumors in adults; they are derived from glial or precursor cells (20). These are a heterogeneous group of diseases with multiple subtypes (20, 21). Glioblastoma multiforme (GBM) is the most common and fatal form of the primary brain tumor, accounting for approximately 60% of all glioma cases (22), whereas low-grade gliomas (LGGs) are the second most common type of glioma in adults (~30%) (22).

In GBM tumor specimens, the presence of C1q does not correlate with CD45 positive leukocytic infiltration (23). Interestingly, C1q appeared to be highly concentrated around the malignant cells and the necrotic debris. Moreover, the serum concentration of C1q, together with critical components of the lectin and alternative complement pathways, appeared significantly increased in GBM patients as compared to healthy controls (23), thus indicating a role for complement activation in the pathogenesis of the GBM.

In the current study, we performed a bioinformatics analysis aimed at investigating whether C1q can serve as a potential prognostic marker for gliomas.

## Materials and Methods

### Oncomine Database Analysis

The expression levels of *C1qA, C1qB*, and *C1qC* genes in gliomas were analyzed using Oncomine (www.oncomine.org), a cancer microarray database and web-based data mining platform for new discovery from genome-wide expression analyses (24, 25). We compared the differences in mRNA levels between normal tissue and cancer. The mRNA expression level in neoplastic tissues, compared to the healthy tissues, was obtained as the parameters of *P* < 0.05, fold change > 2, and gene ranking in the top 10%. Information about the datasets used in this study is summarized in Table 1.

**Table 1:** Characteristics of the datasets used in bioinformatics analysis.

**Datasets**	**Study description**	**Experiment type**
Sun brain	One hundred fifty-seven (157) brain and CNS tumors and 23 normal brain samples were analyzed on Affymetrix U133 Plus 2.0 microarrays. Sample data includes type, grade, and sample name. Corresponding DNA copy number data is available in Kotliarov Brain	mRNA
French brain	Twenty-three (23) anaplastic oligodendroglioma, 4 anaplastic oligoastrocytoma, and 6 normal brain samples were analyzed on Affymetrix U133 Plus 2.0 microarrays. Sample data includes 10q loss of heterozygosity, 19q loss of heterozygosity, 1p loss of heterozygosity, age, chemotherapy response, EGFR amplification, sex, survival after diagnosis, survival after surgical resection, and therapy	mRNA
TCGA brain	Five hundred forty-seven (547) glioblastoma and 10 normal brain samples were analyzed. Sample data includes age, sex, survival, and others. This dataset consists of Level 2 (processed) data from the TCGA data portal. Corresponding DNA copy number data is available in TCGA Brain 2	mRNA
CGGA brain	The CGGA RNA sequence dataset consisted of 325 samples, including 109 grade-II samples, 72 grade-III samples and 144 grade-IV samples. Of the 144 GBM samples, 6 samples were lost to follow-up; therefore, 138 samples were included in the survival analysis. The patients with GBM were followed up every 3 months	mRNA
Rickman brain	Forty-five (45) astrocytoma and 6 normal temporal lobe samples were analyzed on Affymetrix HuGeneFL microarrays. Sample data include type and grade	mRNA
Bredel brain 2	Fifty (50) brain CNS carcinoma samples and 4 normal brain samples were analyzed on cDNA microarrays. Sample data includes disease type	mRNA
Liang brain	Thirty (29) glioblastoma, 3 mixed astrocytoma-oligodendroglioma, 2 oligodendroglioma, 2 normal brain, and 1 normal cerebellum sample were analyzed on cDNA microarrays. Sample data includes type, age, location, primary/recurrent, sex, and survival	mRNA

### UALCAN and CGGA Database Analysis

UALCAN (http://ualcan.path.uab.edu) is a web resource for analyzing cancer transcriptome data, which estimates the effect of gene expression level on the patient survival (26). In addition to the gene expression variation across tumor samples, gene-level correlations with patient survival also feature in UALCAN. Available genomics data from “The Cancer Genome Atlas” (TCGA) project was used for Kaplan–Meier survival analysis to generate survival probability plots (26). The prognostic significance of *C1qA, C1qB*, and *C1qC* expression and survival in gliomas were analyzed by UALCAN. The hazard ratio with 95% confidence intervals and logrank *p*-value were also computed.

The Chinese Glioma Genome Atlas (CGGA) (http://www.cgga.org.cn) is a user-friendly web application for data storage and analysis exploring brain tumors datasets from Chinese cohorts (Table 1) (27). Analyze tool of CGGA was used to browse *C1qA, C1qB*, and *C1qC* mRNA expression profile and to perform survival analysis in specific glioma subtype. The hazard ratio with 95% confidence intervals and logrank *p*-value were also computed.

### Statistical Analysis

Survival curves were generated by UALCAN and CGGA. All results are displayed with *p*-values from a log-rank test. *P*-values < 0.05 were considered significant. Similarly, in the case of Oncomine, the program provided the statistical significance of data (*P*-values).

### Immunostaining

Tissue samples, derived from the neuroepithelial tumors with astrocytic differentiation, presenting different grades (grade-II and -III to GBM, grade-IV), were collected from glioma patients, after informed consent following approval of the ethical considerations by the Institutional Board of the University Hospital of Trieste, Italy.

Gliomas tissue specimens (five patients for each glioma grade, Department of human pathology of the University Hospital of Cattinara, Trieste, Italy) were fixed in 10% v/v buffered formalin and paraffin embedded. For immunostaining, 4 μm-thick tissue sections were de-waxed with two changes of xylene, 10 min each. Slides were then transferred to 100% alcohol, for two changes, 10 min each, and once through 95 and 70% alcohol respectively, for 5 min each. Finally, they were rinsed in de-ionized water, twice for 3 min each. The antigen unmasking technique was performed using Novocastra Epitope Retrieval Solutions pH9 EDTA-based buffer in thermostatic bath at 98°C for 30 min (28). Sections were brought to room temperature and washed in PBS. Subsequently, the neutralization of the endogenous peroxidase with 3% v/v H_2_O_2_ and Fc blocking by a specific protein block (Novocastra, Leica Biosystems) were performed.

For immunostaining, glioma sections were probed with the following primary antibodies overnight at 4°C: rabbit anti-human C1q polyclonal antibody (1:500; Dako), rabbit anti-human C3d polyclonal antibody (1:100; Cell Marque) and rabbit anti-human C4d polyclonal antibody (1:100; Cell Marque). Antibody-Antigen recognition was detected using Novolink Polymer Detection Systems (Novocastra Leica Biosystems, Newcastle) and employing the high sensitivity AEC (3-Amino-9-Ethylcarbazole) as chromogen. Slides were counterstained with Harris Haematoxylin (Novocastra, Ltd) and images were collected using a Leica DFC320 digital camera (Leica Microsystems, Wetzlar, Germany).

For double immunostaining experiments, tissue sections were incubated overnight at 4°C with the following primary antibodies: rabbit anti-human C1q polyclonal antibody (1:500, Dako), mouse anti-human CD68 (1:50, Clone KP1, Dako) and anti-human CD163 monoclonal antibodies (1:100, Clone 10D6, Leica Biosystems). The following secondary antibodies were used: goat anti-rabbit conjugated to Alexa Fluor 488, and goat anti-mouse conjugated to Alexa Fluor 568 (Life Technologies). Nuclei were counter-stained with DAPI (4′,6-diamidin-2-fenilindolo). All the sections were analyzed under Zeiss Axio Scope A1 optical microscope (Zeiss, Germany) and microphotographs were collected using an Axiocam 503 Color digital camera with the ZEN2 imaging software (Zeiss Germany).

## Results

### Bioinformatics Analysis Reveals Higher mRNA Levels of *C1q A, B*, and *C* Chains in Gliomas

**Figure 1:**
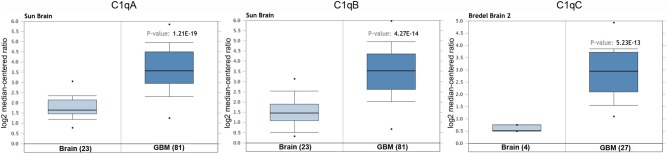
*C1qA, C1qB*, and *C1qC* expression in gliomas. Sun's dataset was used for bioinformatics analysis to explore *C1qA* and *C1qB* mRNAs expression in the glioblastoma multiforme, whereas Bredel 2's dataset was used for bioinformatics analysis to evaluate *C1qC* mRNA. A higher expression of the three chains was detectable in glioblastoma multiforme compared to normal brain tissue. GBM, glioblastoma multiforme.

We initially compared the mRNA levels of the three chains of human *C1q* (*A, B*, and *C*) in normal brain and gliomas using the Oncomine platform. From the analysis performed on several datasets such as Sun's, French's, TCGA's, Rickman's, Bredel 2's, and Liang's, a significantly higher mRNA expression levels for *C1qA, C1qB*, and *C1qC* chains were detected in gliomas (different histotypes and grades) as compared to normal brain tissue (Figure 1, *P* < 0.05; Table 2).

**Table 2:** *C1qA, C1qB*, and *C1qC* expression in gliomas in the datasets used in the current study with Oncomine.

**Datasets**	**C1q chains**	**Brain vs. tumor**	***P*-value**
Sun	*C1qA*	Brain (23) vs. Anaplastic astrocytoma (grade-III) (19) Brain (23) vs. Glioblastoma multiforme (grade-IV) (81) Brain (23) vs. Anaplastic astrocytoma (grade-III) (19)	3.59E-6 1.21E-19 5.22E-6
	*C1qB*	Brain (23) vs. Glioblastoma multiforme (grade-IV) (81)	4.27E-14
French	*C1qA*	Brain (6) vs. Anaplastic oligoastrocytoma (grade-III) (4) Brain (6) vs. Anaplastic oligodendroglioma (grade-III) (23)	8.89E-4 3.59E-6
	*C1qB*	Brain (6) vs. Anaplastic oligoastrocytoma (grade-III) (4)	5.89E-4
TCGA	*C1qA*	Brain (10) vs. Glioblastoma multiforme (grade-IV) (542)	1.63E-8
	*C1qB*	Brain (10) vs. Glioblastoma multiforme (grade-IV) (542)	3.45E-4
Richman	*C1qB*	Temporal lobe (10) vs. Astrocytoma (different grade) (45)	7.87E-4
Bredel 2	*C1qC*	Brain (4) vs. Glioblastoma multiforme (grade-IV) (28)	5.23E-13
Liang	*C1qC*	Brain (2) vs. Glioblastoma multiforme (grade-IV) (30)	2.12E-4

*n, samples number*.

**Figure 2:**
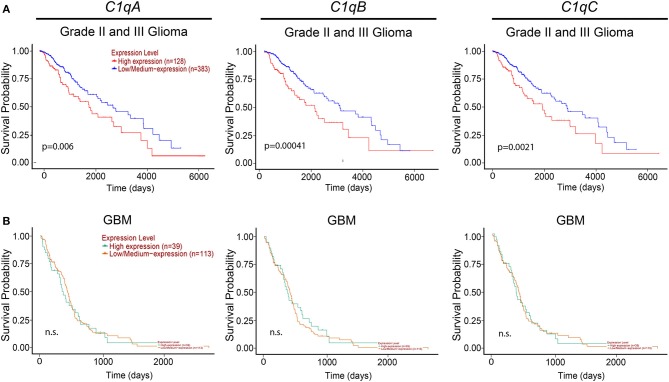
Pathological significance of C1q expression in gliomas. According to the UALCAN database analysis, *C1qA, C1qB*, and *C1qC* mRNAs expression were negatively linked to a survival probability in patients with LGGs (grade-II and -III) **(A)**, whereas no correlation in patients with GBM (grade-IV) **(B)**. LGGs, low-grade gliomas; GBM, glioblastoma multiforme.

We then took advantage of the UALCAN tool to carry out bioinformatics analysis on *C1qA, C1qB*, and *C1qC* mRNA expression levels according to TCGA database. UALCAN tool considers LLGs, grade-II and -III, and high-grade gliomas (HGGs) only grade-IV (or GBM) while the World Health Organization (WHO) considers as LLGs, grade-I and -II, and HGGs as grade-III and -IV. Based on this analysis, as shown in Figure 2, a positive correlation between the mRNA expression of the three chains and the unfavorable prognosis only in LGGs (grade-II and -III) was evident, where the survival probability is indeed reduced (*P* < 0.05). By contrast, no correlation was observed between *C1qA, C1qB*, and *C1qC* mRNA expression and the survival probability in GBMs (grade-IV) (Figure 2).

**Figure 3:**
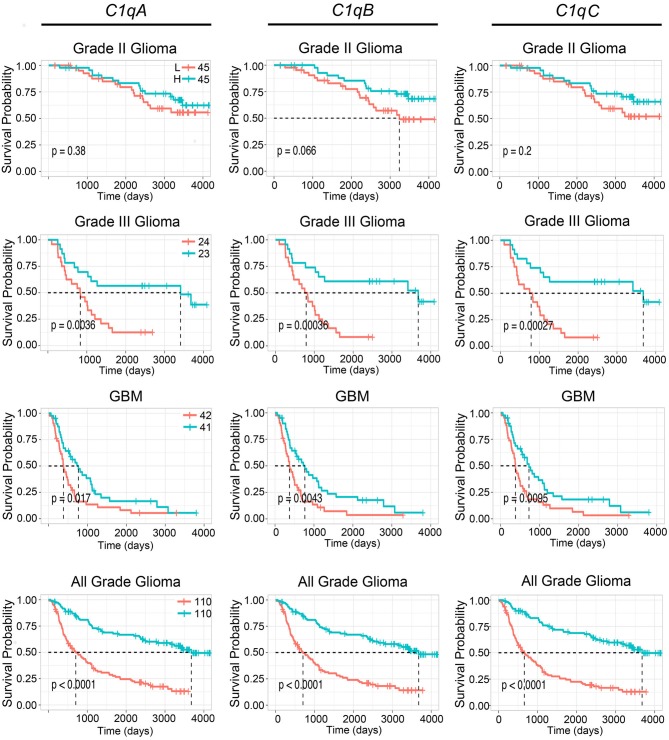
Pathological significance of C1q expression in gliomas. According to the CGGA database analysis, *C1qA, C1qB*, and *C1qC* mRNAs expression were negatively linked to a survival probability in patients with grade-III and GBM. H, high expression; L, low expression.

To further validate these results, we used the CGGA tool to inquire an independent glioma database. Based on this analysis, a positive correlation was found between the mRNA expression of the three C1q chains and the unfavorable prognosis in all WHO grade of gliomas, where the survival probability is indeed reduced (*P* < 0.05) (Figure 3, lowest panels). A similar unfavorable prognostic effect was detected in grade-III gliomas (*P* < 0.05) while no correlation was observed between *C1qA, C1qB*, and *C1qC* mRNA expression and the survival probability in gliomas grade-II (Figure 3). In contrast to UALCAN analysis, a negative prognostic effect was underscored in GBMs (*P* < 0.05) (Figure 3).

### Low- and High-Grade Gliomas Abundantly Express C1q Protein

**Figure 4:**
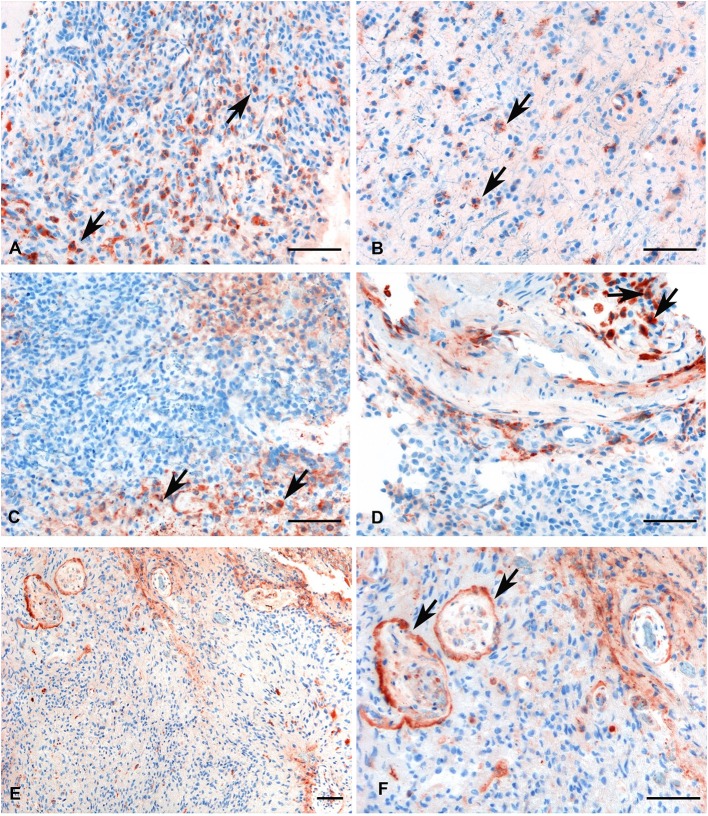
Representative immunohistochemical analysis of C1q in low **(A,B)** and high grade **(C,D)** gliomas showing a high expression in both histotypes regardless of grade. C1q results mainly expressed by macrophages and the vascular stroma (see arrows). C1q expression in the endothelial cells is shown in panels **(E,F)** (see arrows, low, and high magnification). Polymer detection system with AEC (red) chromogen; scale bars, 50 μm.

We investigated the presence and the distribution of C1q in several glioma samples of different grades. As shown in Figure 4, a high expression level of C1q was observed both in LGGs as well as GBM.

**Figure 5:**
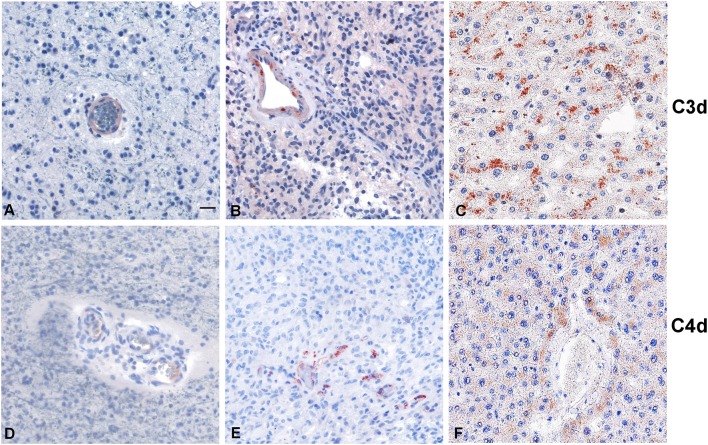
Representative immunohistochemical analysis of C3d and C4d complement activation products in low grade **(A,D)** and high grade **(B,E)** gliomas. C3d and C4d specific staining is segregated in the blood vessels, while the tumor tissues are negative. Liver staining **(C,F)** represents the antigen (tissue) control. Polymer detection system with AEC (red) chromogen; scale bars, 50 μm.

Within the tumor-associated microenvironment, C1q was mainly expressed by monocytoid cells, suggestive of tumor associated macrophages (arrow heads) scattered among the neoplastic cells, which show an increased density around the intra-tumoral necrotic foci (Figure 4C). Moreover, the presence of C1q was detected in association with the vascular stroma; in GBM, it was also expressed in the vascular endothelial cells (Figures 4E,F). C1q deposition was not associated with complement activation, occurring either via the classical or the other pathways, since we failed to observe any C3d and C4d immunoreactivity in our glioma's specimens (Figure 5).

### Infiltrating M2 Macrophages Are Likely Source of C1q in Gliomas

**Figure 6:**
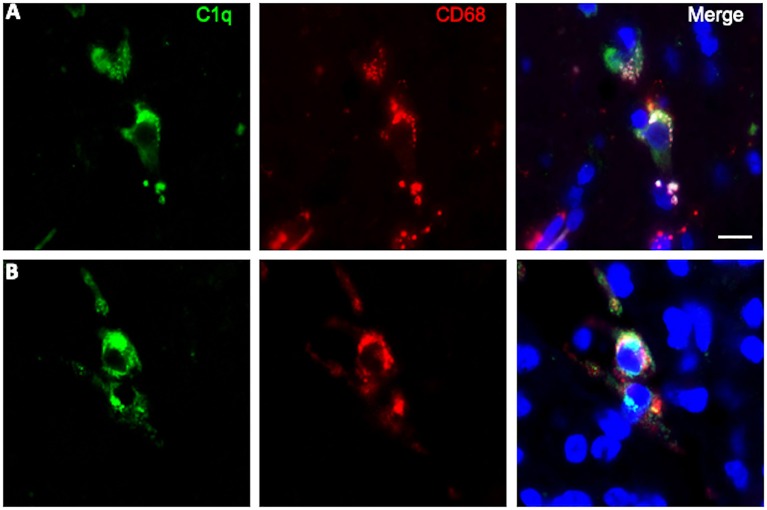
Representative microphotographs of double immunofluorescence for C1q (green signal) and CD68 (red signal) in FFPE sections of low **(A)** and high **(B)** grade gliomas confirming the macrophage nature of C1q expressing monocytoid elements. The cell nuclei were stained with DAPI; scale bars, 10 μm.

**Figure 7:**
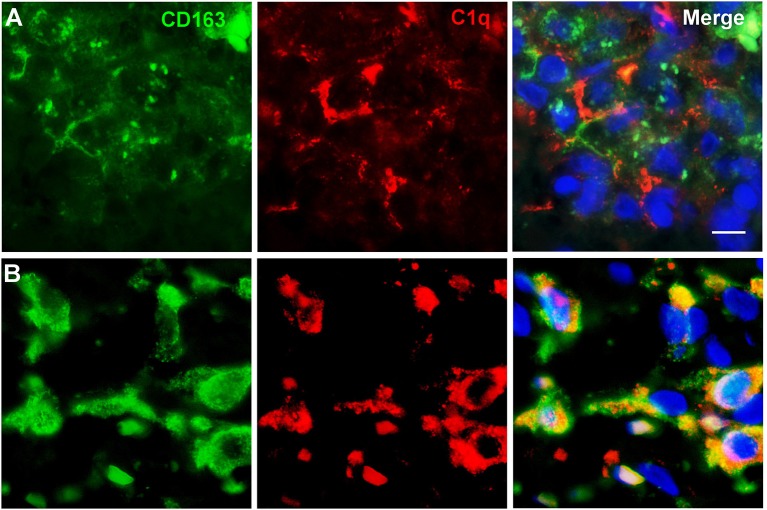
Representative microphotographs of double immunofluorescence for C1q (red signal) and CD163 (green signal) in FFPE sections of low **(A)** and high **(B)** grade gliomas confirming the M2-polarization of macrophages expressing C1q. The cell nuclei were stained with DAPI; scale bars, 10 μm.

To further characterize the cell type infiltrating the tumor and actively involved in C1q synthesis, immunocytochemical experiments were performed on gliomas specimens via staining for C1q and CD68, a specific marker for monocyte/macrophage cell types. A clear co-localization of C1q/CD68 immunoreactivity was detected in both low- and high-grade gliomas (Figure 6), thus identifying the macrophages infiltrating glioma tumor as the main source of local C1q synthesis and secretion. Double labeling experiments were also performed using an anti-CD163, a specific marker for the tumor-promoting M2-polarized macrophages (Figure 7). Under these conditions, it appeared that not only the number of CD163 positive cells, but also the CD163 expression level itself, were increased in high-grade gliomas as compared to low-grade ones. Most of the C1q labeled cells colocalized with CD163, even though a small fraction of infiltrating cells were solely expressing C1q, suggesting that they may correspond to the CD68/C1q co-expressing M1-type of macrophages.

## Discussion

Glial tumors, also called gliomas, are the most prevalent form of adult brain tumor, accounting for nearly 80% of all brain malignancies (22). Based on their histological features and expression of lineage markers, gliomas can be classified into astrocytomas, oligodendrogliomas, ependymomas, and choroid plexus tumors (29). Astrocytomas, which represent almost half of all primary brain and spinal cord tumors, may occur in the brain with a preferential localization in the cerebrum and affect mostly adults, particularly middle-aged men. In 2016, the WHO has redefined this classification scheme by introducing molecular parameters in addition to the well-established histopathological features (20, 21). These new guidelines have allowed classification of all pathological glial entities in four grades, according to histological parameters including nuclear atypia, mitoses, vascular proliferation, and necrosis. According to the grading system, astrocytic neoplasm can be divided into low and high-grade astrocytomas. Low-grade astrocytomas are the least malignant tumors characterized by slow growth and good prognosis being the *pilocytic astrocytomas* (grade-I) and *diffuse astrocytomas* (grade-II), the most frequent types. High-grade astrocytomas are glial tumors presenting a rapid growth with a tendency to infiltrate nearby brain tissues. They are divided into astrocytomas grade-III (*anaplastic astrocytoma*) and grade-IV (*glioblastoma multiforme, GBM*). GBM is the most aggressive and fast-growing malignancy characterized by poor clinical outcome. It can arise in the brain *de novo*, or it can evolve from lower-grade astrocytomas or oligodendrogliomas (29). Recent studies have begun to address the immune signature of the glioma microenvironment and its relationship with prognosis (30). The fact that C1q can be locally synthesized within the CNS and that it is involved in tumor immunology, we wanted to interrogate its relevance in the pathogenesis and prognosis of gliomas.

In the current study, we performed a bioinformatics analysis to unveil whether C1q could serve as a potential prognostic marker for these devastating malignancies. UALCAN queries of the TCGA and the CGGA datasets were analyzed to validate our initial result. It highlighted a significant correlation between high expression level of the three chains of the C1q and poor prognosis in gliomas of diverse grade of malignancy (Figures 2, 3). In particular, while interrogating the CGGA dataset, which allowed distinction between grade-II and grade-III gliomas, a significant correlation was established only for grade-III gliomas (Figure 3). In the TCGA dataset, significance was achieved for the so-called LGGs, which combines grade-II and -III cases (Figure 2). The main contradiction was noticed in GBMs (grade-IV gliomas), where opposite prognostic effects were underscored in relation to the dataset used (Figures 2, 3). One possible explanation for these contradictory observations may originate from the algorithm utilized by the bioinformatics web resources to define high and low expression profile of the genes. Another aspect to take into account is potential differences in the genetic and epigenetic signatures characterizing the two datasets, the TGCA mostly relying on Caucasian patients while CGGA relying on Chinese cohorts. Indeed, it has been proven that substantial variation in glioma incidence and survival are connected, to some extent, to race and ethnicity (31). Finally, it is worth mentioning that GBM exists in two forms, primary and secondary, indistinguishable histologically, but clearly discernible clinically and in terms of molecular signatures (29, 32). Primary GBM is the most common form that occurs mainly in adults over 50 years of age. The genetic profile is characterized by epidermal growth factor receptor (EGFR) overexpression, phosphatase and tensin homolog (PTEN) mutation, p16 deletion, and chromosome 10 loss (32). Secondary GBM derives from a malignant progression of diffuse or anaplastic astrocytomas (grade-II and -III, respectively), and occurs in younger patients, characterized by p53 mutation and a reduced state of heterozygosity in tumor cells (loss of heterozygosity) on chromosome 10q (32). Both datasets used in our bioinformatics analysis did not discriminate between *de novo* and secondary GBM, possibly masking a significant correlation, indeed expected, in those tumors progressing from grade-III gliomas.

There is emerging evidence to confirm that C1q is involved in cancer pathophysiology, being an important modulator of inflammation and cytokine/chemokine/growth factor secretion. What is still quite puzzling is whether and when C1q is protective against or supportive of cancer progression. On one hand, C1q can be detrimental to cancer cell viability via its cell lytic, anaphylatoxin, and opsonin effector mechanisms (33). Alternatively, C1q can exert tumor-promoting functions which are independent of the classical pathway activation. As observed for other types of cancers (10, 15), C1q deposition in gliomas seems not to be correlated with complement activation as we were unable to detect the complement split products C3d and C4d in the cohort of tumor specimens tested (*n* = 5). It is worth noting that our conclusions are based on the use of only five patients' tissue samples that were subjected to immunohistochemistry. This is in contrast to other cancers such as renal and lung cancers, where C4d deposition has been reported (34, 35). The mechanisms underlying such binary features of C1q are likely to be shaped by the type of cancer cells, nature and extent of infiltrating immune cells and their ability to synthesize locally C1q (and/or other complement components), and most crucially, the biochemical nature of the tumor microenvironment.

In the brain, complement components, including C1q, can be locally produced by resident neurons and glial cells, microglia and astrocytes being the major producers (19, 36). Our immunocytochemical data demonstrated that CD68 and CD163 positive infiltrating cells represent the cell types actively synthesizing C1q in the tumor micro-environment (Figures 6, 7). CD68 expression is characteristic of tumor-associated macrophages, whose enrichment in glioma has been associated with poor prognosis (37). CD163 identify the M2-polarized macrophages, which are highly versatile cells known to influence multiple steps in tumor development and invasiveness along with angiogenesis and immunosuppression (38).

These cells in the brain are derived from two different sources: resident microglia and monocytes/macrophages that enter the brain from bone marrow. Even though it is quite difficult to distinguish between these two different cell types due to the lack of available definitive markers, they are recruited by the tumor microenvironment via several gliomas derived chemokines that also contribute to their polarization from a tumor-suppressive to a tumor-promoting phenotype (39). It is interesting to note that C1q has been shown to enhance the secretion and action of these chemokines (40, 41). Therefore, C1q produced and released by microglia/macrophage cells, is expected to promote immunosuppression, thus favoring glioma cell proliferation. This is consistent with our observation linking the expression of the three chains of human C1q with an unfavorable prognosis in grade-II and -III gliomas (LGGs) in TCGA dataset and in grade-III gliomas in CGGA dataset, respectively (Figures 2, 3).

We also observed a high degree of deposition of C1q in the perivascular stroma as well as on endothelial cells belonging to the tumor vasculature. Developing gliomas require an increased nutrient supply and hence trigger neovascularization via the release of angiogenetic factors by cancer cells. C1q itself promotes angiogenesis through its globular heads (7). Massive angiogenesis is induced by regions of hypoxia within the tumor that are not only loci generating damage-associated molecular patterns (DAMPs), recognized by C1q but also privileged sites for glioma stem-like cell (GSC) settlement (42). These cells are quite dangerous since they maintain tumor growth through self-renewal amidst a supportive microenvironment. C1q, in this context, has been shown to participate in GSC maintenance and expansion via the activation of the canonical Wnt signaling cascade through its binding to Frizzled-receptor (43).

Our bioinformatics study, based solely on mRNA expression dataset and therefore further requiring a validation at the protein level, underlines how complex, multifaceted and yet feebly understood is the differential role of C1q in tumor progression or suppression. In conclusion, C1q plays a fundamental role in the pathogenesis of gliomas, but further investigation is required for its use in the clinic as a prognostic marker.

## Data Availability Statement

Publicly available datasets were analyzed in this study. This data can be found here: TCGA-GBM, TCGA-LGG (https://tcga-data.nci.nih.gov/tcga/), and CGGA (http://www.cgga.org.cn).

## Ethics Statement

This study was carried out in accordance with the recommendations of government guidelines, and approved by the CEUR (Comitato Etico Unico Regionale, FVG, Italy; number 34/2016). All subjects gave written informed consent in accordance with the Declaration of Helsinki.

## Author Contributions

AMan, PZ, and RB: conceptualization and writing–original draft preparation. AMar and BB: methodology. AMan: software and visualization. DB: validation. FZ: formal analysis. BB and DB: investigation. AMan, VR, DI, and FZ: resources. CA: data curation. PZ, CA, and UK: writing–review and editing. RB: supervision and project administration.

### Conflict of Interest

The authors declare that the research was conducted in the absence of any commercial or financial relationships that could be construed as a potential conflict of interest.
